# Protists in the Insect Rearing Industry: Benign Passengers or Potential Risk?

**DOI:** 10.3390/insects13050482

**Published:** 2022-05-21

**Authors:** Edouard Bessette, Bryony Williams

**Affiliations:** 1 Living Systems Institute, Biosciences, College of Life and Environmental Sciences, University of Exeter, Exeter EX4 4QD, UK; e.bessette@exeter.ac.uk; 2 Department of Plant and Environmental Sciences, University of Copenhagen, 1871 Copenhagen, Denmark

**Keywords:** mass rearing, edible insects, protists, microsporidia, insect diseases

## Abstract

**Simple Summary:**

As human populations grow and the climate crisis deepens, humans will need to look to alternative sustainable sources of protein. The insect rearing industry is now rapidly growing to generate more sustainable sources of food and feed, and, as it does so, there will be an urgent need to better understand the role that microorganisms play in both maintaining insect health and generating disease. Protists are microbes that are neither viral, bacterial nor fungal and, therefore, are sometimes overlooked when considering microbial fauna. In this paper, we review the literature on protists that have been uncovered within insects that are being considered for rearing as food and feed. We discuss what is known about how they interact with hosts, how they may affect industrially reared insects in the future and which tools now need to be developed to better study them.

**Abstract:**

As the insects for food and feed industry grows, a new understanding of the industrially reared insect microbiome is needed to better comprehend the role that it plays in both maintaining insect health and generating disease. While many microbiome projects focus on bacteria, fungi or viruses, protists (including microsporidia) can also make up an important part of these assemblages. Past experiences with intensive invertebrate rearing indicate that these parasites, whilst often benign, can rapidly sweep through populations, causing extensive damage. Here, we review the diversity of microsporidia and protist species that are found in reared insect hosts and describe the current understanding of their host spectra, life cycles and the nature of their interactions with hosts. Major entomopathogenic parasite groups with the potential to infect insects currently being reared for food and feed include the Amoebozoa, Apicomplexa, Ciliates, Chlorophyta, Euglenozoa, Ichtyosporea and Microsporidia. However, key gaps exist in the understanding of how many of these entomopathogens affect host biology. In addition, for many of them, there are very limited or even no molecular data, preventing the implementation of molecular detection methods. There is now a pressing need to develop and use novel molecular tools, coupled with standard molecular diagnostic methods, to help unlock their biology and predict the effects of these poorly studied protist parasites in intensive insect rearing systems.

## 1. Introduction

The human population is expected to grow to ~11 billion by 2100, placing pressure on an already fragile food production system [1]. Coupled with a need to reduce water usage and greenhouse gas production, it is expected that this will result in more reliance on alternative sustainable sources of protein. Aquaculture invertebrates and industrially reared insects are set to become an important source of protein for human consumption and for animal feed [2]. As the insects for food and feed industry grows, a new understanding of the industrially reared insect microbiome is needed to better understand the role that microorganisms play in both maintaining insect health and generating disease.

Since 2016, insects have been recognised as husbandry animals (like poultry and cattle) by the European Federation of Animal Science (EAAP). The insect rearing industry is rapidly growing in Europe to meet the global demand for improved foods with regard to societal and environmental concerns. In 2017, 6000 tons of insects were produced for animal feed in Europe alone [3]. The global production of insects is estimated to reach 100,000 tons by 2023 and a 10-fold increase is foreseen for 2030 [4].

To date, the European regulation authorises the use of insect proteins in feed for aquaculture, poultry and swine animals from eight insect species: the common housefly (*Musca domestica*), the black soldier fly (*Hermetia illucens*), the mealworm (*Tenebrio molitor*), the lesser mealworm (*Alphitobus diaperinus*), the silkworm (*Bombyx mori*), the house cricket (*Acheta domesticus*), the banded cricket (*Gryllodes sigillatus*) and the field cricket (*Gryllus assimilis*) [5]. Some other species are produced as pet food, such as the desert locust (*Schistocerca gregaria*) and the greater wax moth (*Galleria mellonella*), and others as human food, such as the larvae of *T. molitor* [2,5]. Crickets and grasshoppers/locusts can also be used as food in Europe. The house cricket, *A. domesticus*, is used for protein bars, snacks and pasta, and the grasshopper *L. migratoria* is sold as flavoured snacks (dried or frozen) [3,5]. The black soldier fly (BSF) has also shown potential as human food, even if, currently, this species is only raised for feed applications in the EU.

Insect farming occurs under high-density populations in artificial environments, and, under these conditions, infectious diseases can be easily spread. For example, in the 1990s, a virus wiped out the house cricket production at Monkfield, a UK based company [6]. To provide an idea of the production scale, every week, Monkfield produces about four million crickets and locusts. As locusts are typically reared in bins without lids, contamination via a passive vector, such as house flies or other insect intruders, is quite feasible [6].

This example highlights the need to better understand the diversity of pathogens that commercially reared insects may be susceptible to and how they may affect insect health.

Often, in microbiome studies, the roles and identities of microeukaryotic pathogens other than fungi are generally poorly studied [7,8,9,10] in spite of their potential to cause serious disease in intensively reared invertebrates. Microeukaryotic pathogens can often exist as latent, benign infections, but, in the past, it has been shown that these can contribute to serious ill health, low weight or changes in life cycle in commercially reared invertebrates when other stressors are present [11,12].

In some cases, protistan pathogens have been originally described from a mass rearing system, such as microsporidia pathogens isolated from *Drosophila*, mosquito, locust or cricket lab colonies, honeybees or farmed shrimp [11,13,14,15,16,17,18], rather than from wild insects. Moreover, it is possible that some of those pathogens emerge or cross species boundaries only when the hosts are under that stress of a mass rearing system. This emergence risk makes it crucial that we try to better understand the diversity of protist pathogens, how to identify them and to make predictions about how they may affect intensively reared insects.

Here, we will review key non-fungal microbial eukaryotes identified to date within insects that are currently being reared commercially. We discuss their potential to cause serious disease in the context of their relationship to other known animal pathogens, reviewing the data available for each in terms of pathogenicity.

## 2. Protist Parasites, Including Microsporidia, within the EU Reared Insect Species

Prokaryotic microbes, fungi and viruses are actively being studied as potential pathogens of intensively reared insects [19,20]. The Microsporidia, a group of intracellular pathogens that are considered part of a sister group to fungi [21], are also intensively studied in invertebrates; however, other single-celled eukaryotic parasites are less well-studied [22].

The protists represent an incredible diversity of cellular forms and biochemistries with multiple diverse groups that have the capacity to infect animals and, in some cases, cause serious disease. Inevitably, these include insect hosts, and multiple protist groups have been found to cause infections in insect species reared for food and feed, either as natural or experimental infections (see Supplementary Tables S1 and S2). Moreover, these insect–protist interactions reflect the whole spectrum of symbioses. Indeed, they range from commensalism, where the host only provides a spatial niche for the protist symbiont with no harm for the host, to parasitism, whereby the parasite uses host energy for its own development and reproduction, and, eventually, mutualism, where the symbiont and host are both receiving benefits from the interaction (Box 1). Typically, protist parasites start their parasitic life cycle when they or their environmental spores come into contact with or are ingested by a susceptible insect host [12,20]. The infective stages will then infect specific tissues in insect larval instars and adults.

Box 1 **General definitions.**

**Protist:** Historically called protozoan, the term protist is typically used to describe all
eukaryotes other than fungi, animals and plants [9]. **Symbiont:** The definition of symbiont is any
organism that establishes a long-lasting and durable relationship with
another organism. In the scientific field, a symbiont is often cofounded with
a true mutualist, where a mutualism interaction brings benefits to both
linked organisms [23]. **Parasite:** In this review, a parasite is
defined as an organism that must live at the expense of a host for its own
benefits and reproduction, where the parasite inflicts a direct cost to the
infected host by reducing its fitness (e.g., reduced fecundity, growth,
survival or mating success) [24]. However, in certain ecological context, being
parasitised can provide benefits to the host [24].**Commensal:** A commensal is a symbiont that
lives at the expense of another organism without inducing any pathogenic
effect to it. **Heteroxenous and homoxenous (or monoxenous):**
These terms define parasites that either complete their life cycle within a
single host (monoxenous) or have a life cycle that involves at least two host
species (heteroxenous). For heteroxenous parasites, the different hosts can
be distinguished as intermediate and definitive hosts. The definitive host is
the organism that supports the adult or sexually reproductive stage of a
parasite. **Facultative and obligatory parasites:** Facultative
parasites are organisms that are generally free living but can parasitise a
host through their life cycle, whereas an obligate parasite cannot complete
its life cycle without the host.

### 2.1. Amoebozoa

**Figure 1:**
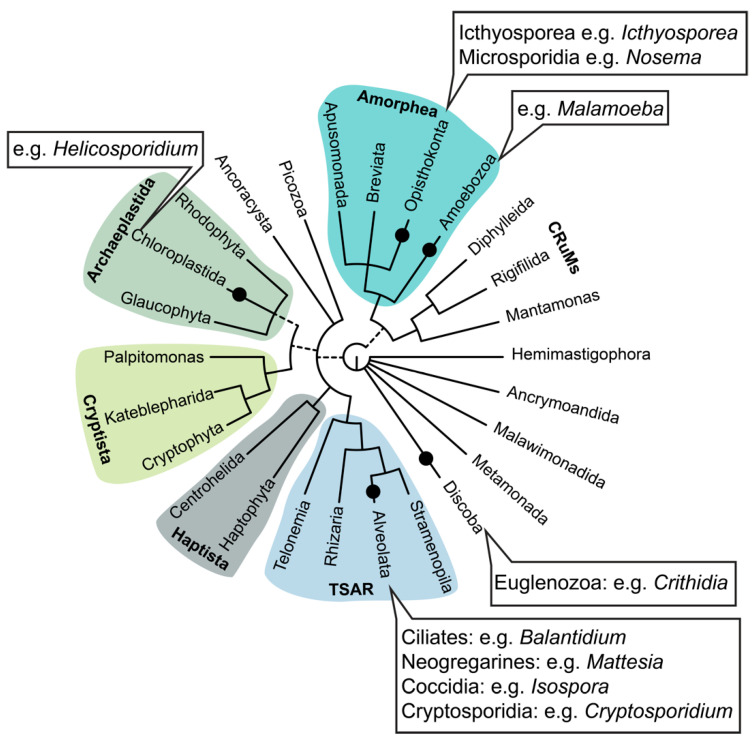
The diversity of protists found in association with insects (black dots). Tree of relationships between major eukaryotic groups; relationships redrawn from Burki et al. 2020 with dashed lines indicating uncertainty about monophyly of groups [25].

Organisms within the Amoebozoa group form a major lineage of eukaryotes as part of the supergroup Amorphea, which comprises, amongst other organisms, animals and fungi (Figure 1). The Amoebozoa includes unicellular eukaryotes that possess pseudopodia for their motility and ingestion, but also some slime moulds, which also have multicellular stages [25,26]. Phagocytosis is the primary mode of nutrition of amoeboids, and their prey include bacteria, algae, smaller protists and yeast. As such, these organisms are important in aquatic and terrestrial environments, where they constitute a link in the food webs between microbes and bigger organisms, such as invertebrates [26]. Arguably, the best-known amoebozoan species is *Entamoeba histolytica,* which is known worldwide because of its medical impact. It invades the human gut and causes serious diarrhoea that can lead to death [26]. *E. histolytica* is estimated to infect 50 million people worldwide and induce 100,000 deaths annually [26].

Amoebae associated with insect hosts are found in the clade Archamoebae, which is constituted of free-living or endobiotic amoebae and amoeboflagellates (i.e., organisms with both flagellates and amoebae in their life cycle) [26]. They have a distinctive hyaline (transparent) cytoplasm and bulging pseudopodia. All Archamoebae are microaerophilic and have remnants of mitochondria [26,27].

The name Archamoebae was introduced by Cavalier-Smith in 1983 [27] and is currently used as a class within the Amoebozoa group [28]. The Archamoebae is currently composed of four main clades, the entamoebae, pelomyxids, mastigamoebids and *Rhizomastix*, with *Tricholimax* sometimes treated as a fifth clade or as an *incertae sedis* genus. Nevertheless, phylogenetic analysis splits the group into entamoebids, with the genus *Entamoeba*, and pelobionts (pelomyxids, mastigamoebids and *Rhizomastix*) [28].

Archamoebae associated with insect hosts have mostly been found from the genera *Entamoeba*, *Dobellina* (Entamoeba group), *Endamoeba* and *Endolimax* (Pelobiontida group) [12]. They are often considered as commensals and have been isolated from the digestive tracts of cockroaches, termites, crane flies and coleopteran [29,30].

#### Morphology, Host Range and Life Cycle

Until recently, only six species have been reported as entomopathogenic, with two species isolated from grasshoppers, *Malamoeba locustae* (formerly *Malpighamoeba*) and *Malamoeba indica* (potentially an isolate of *M. locustae*), one from bark beetles (*Malamoeba scolyti*), honeybees (*Malpighamoeba mellificae*), fleas (*Malpighiella refringens*) and a bristletail (*Vahlkampfia* sp.) [12]. These species are known to form resilient uninucleate cysts in the environment and to infect the host Malpighian tubules and midgut [12]. The conditions that lead to the formation of cysts are not known in these amoebae; therefore, they may potentially spend extended periods as trophozoites only [12]. Nevertheless, encystation is an important part of amoebozoan life cycles, whereby dormant cysts can survive unsuitable conditions, such as a lack of adequate food or low humidity [12].

Excystation and return to an active state occurs when environmental conditions improve. The amoeba’s trophozoite forms (i.e., active, feeding stages) are typically uninucleate but can be binucleate or multinucleate cells. They range from 7 to 11 µm and contain many mitochondria-like organelles [31]. In contrast to many species that have shells or flagella, the entomopathogenic species are naked amoeboids without flagella [12].

**Figure 2:**
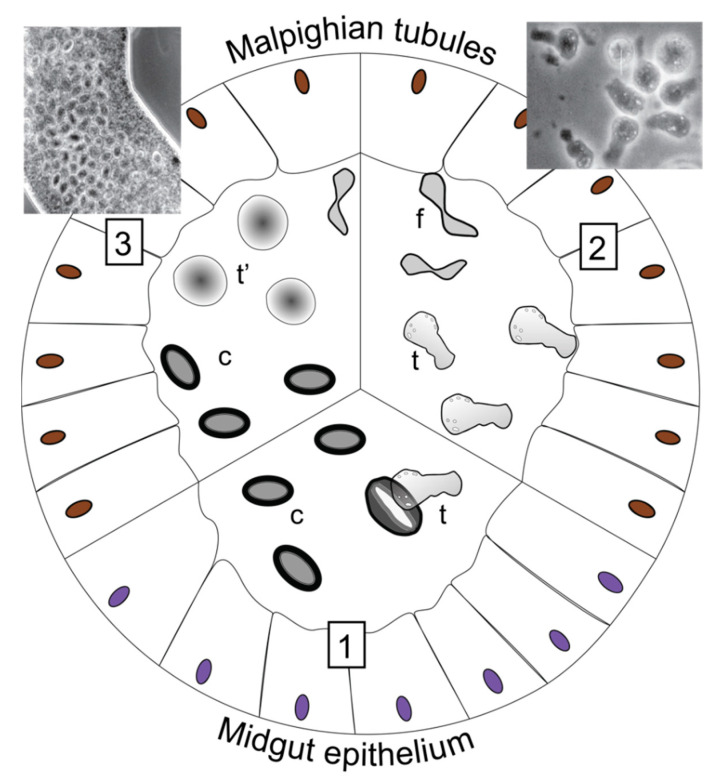
Life cycle of amoebae based on *Malamoeba locustae*. (**1**) Ingestion of infective cysts (c) and excystation with release of trophozoite (t). (**2**) Then, trophozoites penetrate the midgut or gastric caeca epithelial cells and invade the Malpighian tubules. They reproduce by binary fission (f). (**3**) While trophozoites mature, they become rounded (t’) and their cell membrane is surrounded by the cyst wall material. Phase contrast light micrographs show microscopy of (left) infected Malpighian tubules with numerous mature cysts and (right) trophozoites and two immature cysts (reproduced with permission from Lange and Lord 2012 [12] Elsevier Science & Technology Journals).

If we take the life cycle of *Malamoeba locustae* as a typical example, insect hosts become infected when cysts are ingested and pass through the foregut (Figure 2). It is still unclear how the excystation proceeds, but it is thought that the cyst’s wall might be digested in the gut or trophozoites may emerge from the cyst through a lateral break [12]. Thus, trophozoites emerge (one per cyst) and stay around the midgut epithelial cells [31]. Trophozoites are rarely seen within the cell, where they seem to degenerate, but rather stay in the basement membrane of epithelial cells [32]. Then, they leave the midgut epithelium and enter the Malpighian tubules at their junction with the gut (after 5–6 days of infection) [32,33]. Here, they feed by attaching to the brush border of the tubule cell, and eventually reproduce [32,33]. Their reproduction is generally asexual by binary fission [12]. As they feed and multiply, the trophozoites destroy the tubule cells, leaving only the basement membrane [31]. The production of new protective cysts happens 14 days post infection [32]. Within the desert locust *S. gregaria,* the cysts of *M. locustae* measure between 9 and 12 µm in length and 5 to 7 µm in width but can be bigger when recovered from frass [33].

Although *M. locustae* is the best-studied of the entomopathogenic amoebae, many details of some parts of the life cycle still remain unclear, particularly, for example, the mechanism of trophozoite feeding. It seems that trophozoites phagocytose small pieces of the apical brush of the Malpighian tubules [34], but this was not confirmed by other studies [32].

#### Interactions with Hosts

*M. locustae* is a species of potential interest for the insect rearing industry as an amoeba infecting the Malpighian tubules of numerous orthopterans [35], and that is known to cause great damage to laboratory cultures of grasshoppers and locusts [36]. This species is, to date, the most-studied entomopathogenic amoeba; however, most of the studies on this organism come from the 1970s and 1980s [32,33,34,35,37,38,39].

*M. locustae* has been found to infect the midgut, the paired gastric caeca and the Malpighian tubules [34,39]. Malpighian tubules play an important role in the insect osmoregulation and the excretion of foreign chemical substances, mediated in part by the P-glycoprotein, which is expressed at the apical brush of the tubules [40]. The infection damages the tubules’ epithelium and its apical brush [34,36,41,42], impairing secretion and excretion processes [40]. It has recently been shown in infected *S. gregaria* that *M. locustae* damages the Malpighian tubules, inducing an increase in the tubules’ fluid secretion due to an enlargement of the tubules, and a malfunctioning of the P-glycoprotein dependent detoxification caused by the brush disruption [43]. These effects on the tubules’ physiology cause greater energy costs for the insect in terms of fluid reabsorption and can lead to premature death [43], exacerbated by a reduction in feeding of the infected host [36].

Another study showed that *M. locustae* consumes the fat bodies of infected female grasshoppers (*Melanoplus bivittatus*) [44]. Although the lipid content of eggs from infected female hosts was not impacted, the levels of the eggs’ unsaturated fatty acids were different; inducing a higher cost to the female host metabolism to maintain a viable fatty acids level for its eggs [44].

Like most entomopathogenic protists, *M. locustae* does not induce immediate severe disease but leads to a chronic disease characterised by a general debilitation and a reduction in the host’s fitness [12]. However, within heavily infected hosts, some symptoms can be visible to the naked eye, such as dark melanic spots, loss of appetite and premature death [12,34,36,38].

Interestingly, within natural locusts and grasshopper communities, *M. locustae* susceptibly has been hypothesised to be associated with diet. In a study by Abdel Rahman et al. (2015), only one out of thirteen host species was found to be infected, and it was hypothesised that some species acquired an immunity associated with feeding on the plant *Portulaca oleracea* (Portulacaceae), which contains flavonoids with potential anti-protist properties [45].

Hosts coming from artificial environments tend to be more frequently infected [36,46], where the crowded conditions would be more suited for the transmission of Amoebozoa. Indeed, the sole route of infection is through oral ingestion of cysts, and locusts and grasshoppers tend to cannibalize dead or moribund individuals. Infections of grasshoppers and locusts by *M. locustae* in rearing facilities have been reported from various locations, alluding to a broad global distribution [37,39,47].

### 2.2. Apicomplexans

The Apicomplexans represent a hugely diverse clade of obligate parasites, with more than 6000 species known to infect invertebrates and vertebrates. The Apicomplexa are separated into five major lineages: the Cryptosporidia, which are thought to be the most basal lineage; the piroplasmids, haemosporidia and coccidia (sometimes referred to as the ‘core apicomplexa’) and the gregarines [48].

Apicomplexans are characterised by the infectious stage called ‘zoite’, which has a particular anterior apical complex used for host cell invasion and is composed of specific structural and secretory organelles. The apical complex is the defining apicomplexan feature and a key adaptation to parasitism, but there are also many other diverse ways in which the Apicomplexa exploit their hosts [49]. Apicomplexan life cycles are complex, combining sexual (gamont to zygote stages) and asexual (merogony or schizogony) reproduction. They produce an environmentally resistant stage that can stay in the soil or water for months or years, whereas the zoite motile stages are banana-shaped uninucleate cells that move by gliding [49]. Apicomplexans are often intracellular and more frequently epicellular parasites, usually infecting the gut tissues, Malpighian tubules and fat bodies of insect hosts [12,49]. Some species also have stages that develop in the insect haemocoel [49].

The relationships of certain species of Apicomplexa to their hosts have been well-studied due to the fact that they are aetiological agents of many serious human diseases. This has revealed a variety of adaptations to host cell manipulation, including reorganisation of host organelles, acquisition of host cell nutrients and host immune evasion amongst others [50,51,52]. Cell invasion is typically composed of four phases: (i) first host contact without orientation, (ii) attachment followed by apical reorientation, (iii) induction of the parasitophorous vacuole and (iv) transfer of the parasite into the vacuole [49]. The invasion of the host cell is possible thanks to sequential secretions of molecules that come from the secretory organelles (within the apical complex), such as the micronemes and the rhoptries. Once the zoite has entered the host cell, the parasitophorous vacuole provides protection from the host immune system [49].

In the wild, apicomplexan species tend to have a low pathogenicity within their insect hosts [49]. However, under intensive farming conditions or after the infection of a new susceptible host, apicomplexans might induce a high morbidity and mortality [49].

#### 2.2.1. Coccidia

Coccidians are intracellular and monoxenous or heteroxenous parasites of vertebrates and invertebrates. They have complex life cycles, passing through different stages that allow them to persist in diverse locations within their hosts [49]. Coccidia are mostly parasites of vertebrates, with less than 1% of the described species infecting insect hosts. Even if some coccidians are only transmitted by insect vectors (mechanical transmission), there are eight known Coccidia genera that possess an insect as definitive host [12].

Coccidians, also called haemogregarines, are similar to neogregarines (see below) in the size of vegetative stages, having a merogony phase and lacking a mucron or epimerite (structures that anchor the parasite to the host cell) [12]. However, most recent phylogenies place coccidia within a group of ‘core apicomplexa’, including Haemosporidia and Piroplasmids [48].

##### Morphology, Host Range and Life Cycle

*Adelina* is the only entomopathogenic coccidian genus that is well-studied within insect hosts. *Adelina* species are known to infect Coleoptera, Lepidoptera, Orthoptera, Diptera, Embioptera and Blattaria and thus are a potential threat to commercially reared insect species. They are cosmopolitan and are commonly encountered in surveys of insects in grain facilities [53,54,55,56], where *Adelina castana* and *A. picei* are known to infect the Tenebrionidae *Tribolium castaneum* and *Alphitobius piceus,* respectively [53]. *A. tribolii* is another species that has been reported once to infect both Tenebrionidae *T. castaneum* and *T. confusum* [57]. *Adelina grylli* is known to infect the cricket *Gryllus bimaculatus* [58].

The main transmission pathway for coccidian parasites is via ingestion of contaminated food. Some species develop within the gut and disseminate oocysts through the host faeces, while others that inhabit the fat bodies are transmitted and disseminated when predators, cannibals and scavengers consume the hosts’ tissues [12].

**Figure 3:**
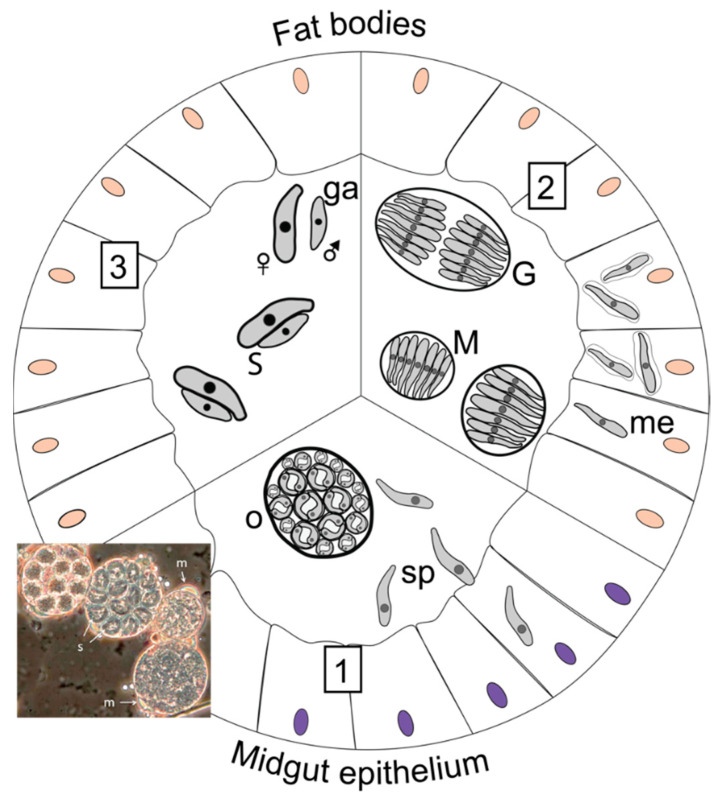
Generalised life cycle of *Adelina* spp. (**1**) Ingestion of an oocyst containing sporocysts (o) and release of sporozoites (sp), which penetrate the epithelium cells (phase-contrast microscopy image shows *Adelina mesnili* oocysts, with sporozoites (s) in sporocysts and microgametocytes (m) indicated, reproduced with permission from Lange and Lord 2012 [12] Elsevier Science & Technology Journals). (**2**) Merogony (M) taking place in fat body cells with meronts protected by a parasitophorous vacuole (me), and, after subsequent merogony, initiation of gametogony (G). (**3**) Sexually differentiated gametes (ga) and their attachment, or syzygy (S). The syzygy leads to the formation of a gametocyst around the gametes, which, once fertilised, will produce a new zygote/oocyst.

The life cycles of *Adelina* spp. are very similar to those of neogregarines [12]. The infection begins with the ingestion of an oocyst containing sporocysts by a suitable host. Each oocyst can release three to twenty spherical sporocysts. Each sporocyst can, in turn, release two vermiform sporozoites that penetrate the midgut epithelium (Figure 3).

The sporozoites undergo some morphological reorganisation after entering the host cell, becoming vacuolated and indistinguishable from merozoites stages. This stage is typical of coccidian motile stages in having an apical complex [58]. The subsequent merogony phase typically occurs within fat body cells of the insect host [59]. The meronts of *A. tenebrionis* were detected 19 days post infection [60] and are typically enveloped by a parasitophorous vacuole that protects them from the host immune system [58]. *Adelina* species develop slowly within the host and some species may require 46 days until the first appearance of oocysts [60], while, in other species, they may appear after 10 days of infection [59].

The size of unsporulated oocysts can measure between 17 and 36 µm in diameter, with a reflective cytoplasm, under light microscopy [53,58]. The infectious stage sporozoites are vermicular and measure around 10 µm in length and 2.5 µm in width, whereas the proliferative stage ‘merozoites’ are slightly bigger (16–18 µm long and 3–4 µm wide) (Figure 3) [12].

##### Interaction with Hosts

The mechanisms of host exploitation and disruption by entomopathogenic Coccidia are not well-characterised, particularly those that infect potentially commercially important insects, but the effects of *Adelina* parasites on *Tribolium* have been studied. The growth rates in infected and uninfected colonies were comparable; however, there was a lag in developmental stages in infected insects (i.e., more adults and fewer larval stages in non-parasitised populations) [57]. Whilst these differences in development are minor within a research setting, they may translate to crucial differences in protein production and profits in an industrial setting.

#### 2.2.2. Gregarinida

Gregarines are poorly studied but represent the main and the most abundant group of Apicomplexa that infect invertebrates [61]. Gregarines are monoxenous parasites with 1800 named species. Most of the species described are from the order Eugregarinida (around 1700 species), and most of them have an insect host [49]. Eugregarines have the simplest life cycle among the apicomplexans, which is composed of a gametogony (the sexual phase) and a sporogony (the asexual phase) that produce the environmental stage, where only a few species undergo a merogony. These organisms are extracellular parasites; they attach to the host cell via their apical extremity by special anchoring structures, either a mucron or an epimerite depending on species [49]. This attachment to the host cell also functions in feeding for the parasite by allowing uptake of the host cytoplasm. In contrast to the Eugregarines, the Neogregarines (*syn*. schizogregarines) can develop intracellularly in the host tissues [12].

The phylogenetic relationships between major apicomplexan groups are not fully resolved, but recent phylogenomic analysis suggests that gregarines may be a sister group to a number of lineages dubbed by some the ‘core Apicomplexa’, branching after the divergence of Cryptosporidium [48].

##### Morphology, Host Range and Life Cycle

**Figure 4:**
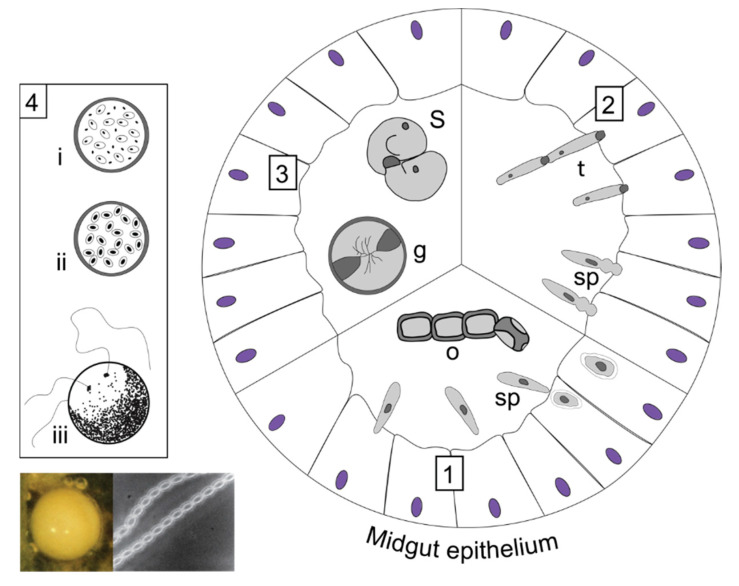
Gregarine life cycle, based on *Gregarina cuneata* in *Tenebrio molitor* [63]. (**1**) Ingestion of oocysts (o) that release sporozoites (sp), which undergo intracellular growth within the midgut epithelium cells. (**2**) The sporozoite growth then continues extracellularly, attached to the epithelium by its epimerite. Trophozoites (t) then form reproductive associations. (**3**) When trophozoites become mature, they undergo a syzygy (S), forming a reproductive gametocyst (g) that is shed into the environment in the host’s faeces. (**4**) Gametogony and fertilisation occur within the expelled gametocyst (i) and form zygotes that become oocysts (ii). Mature gametocysts disrupt releasing infective oocysts into the environment (iii). The oocysts are emitted through sporoducts in chains (iii) [63]. Stereomicroscope image (left) shows a gametocyst in host faeces and phase contrast microscope image (right) shows an oocyst chain (reproduced with permission from Lange and Lord 2012 [12] Elsevier Science & Technology Journals).

A eugregarine infection begins when a susceptible host ingests an oocyst that contains the infectious sporozoite stages. The released sporozoites then develop into large trophozoites, or ‘trophonts’, which attach to the host cell via either a mucron or epimerite [12,49]. The trophonts increase in size and, when they detach from the cell by rupturing of the mucron (or epimerite), they become gamonts. Gamonts can remain in the gut lumen and keep growing before associating by pairs, a process referred to as ‘syzygy’ (Figure 4). Pairing of the trophozoites leads to the encystment of gamonts or ‘gametocysts’. The formed gamonts will produce, after the union of gametes, sporulated oocysts (also called sporocysts), which eventually contain the infective sporozoite stages. Eugregarines are known to possess sporocysts that contain eight sporozoites [49]. After leaving the host via the faeces, the gametocysts released in the environment will initiate a new infection *per os*. The oocysts are freed from the gametocysts by simple rupture or dehiscence through sporoducts, and, eventually, the oocysts, in turn, release the infectious stage’s sporozoites [12].

In the case of the neogregarines, once the tissues are infected by the sporozoites, they multiply through several merogony phases. Compared to eugregarines, the merozoites stages lack a mucron and infect other tissues, such as gonads or fat bodies. Neogregarines usually produce smaller gamonts than eugregarines, and each sporocyst contains eight sporozoites. The oocysts of neogregarines, formed within gametocysts, are ovoid or lemon-shaped, with thick, layered walls and distinct plugs at both poles [12].

Gametocysts are the initial stages observed in an early insect infection; those stages are usually white or yellowish spheres enveloped by a thick, translucent hyaline coat referred to as ectocyst. They can sometimes be visible to the naked eye [12].

They usually develop in cavities of their hosts, such as the digestive tract, the body cavity and the reproductive system. Identification of these parasites is mainly based on morphological traits; however, molecular studies on insect eugregarine species have emerged relatively recently [62].

##### Interactions with Hosts

The absence of merogony in the life cycle of eugregarines suggests that the intensity of infection (i.e., number of parasites within the host) is directly proportional to the number of viable gametocysts or oocysts ingested by the host [12]. Therefore, the key factor governing the intensity of disease caused by the eugregarines is the parasite burden. Several lines of research suggest that eugregarines can fluctuate along the symbiotic spectra between mutualism, commensalism and parasitism [64].

Even if Eugregarines do divert host nutrients for their own benefit, because of their light parasitic burden, they do not induce damage to the host. However, conditions such as those used in mass rearing of insects could lead to heavy gametocyst release and high parasite loads [12]. Under these conditions, Eugregarines could occlude the host midgut, inducing nutrient depletion and a severe slowing of development with high mortality, as has been shown with *Gregarina cuneata*-infected *Tribolium castaneum* [65].

Within the yellow mealworm *Tenebrio molitor*, the symbiotic spectrum of gregarines is wider, where the infection can have a positive effect on host development, increasing fitness and longevity [66,67]. However, *Gregarina niphandrodes* infecting *Tenebrio* adults negatively impacts their longevity when highly infected [68], and *G. cuneata* has been shown to destroy *Tenebrio* gut epithelial cells [69].

Another eugregarine species found in orthopterans, *Gregarina garnhami*, was found in 38.5% of the species of grasshoppers collected directly from the environment (around five species out of thirteen); however, there is no information on the potential pathological effects on hosts [45].

Neogregarines tend to be more pathogenic than Eugregarines since the merogony stage provides massive proliferation capacity [12]. This order contains six families, which are all insect parasites [49]. Common species are *Mattesia dispora*, *M. oryzaephili*, *M. trogodermae* and *Farinocystis tribolii*, all of which infect insect pests of stored grains, fruits and nuts [12]. *M. dispora* induces mortality within the red flour beetle, *T. castaneum* [70]. In lepidopterans, *M. dispora* has also shown a high virulence, with the destruction of fat bodies leading to host death [69]. *F. tribolii* is a common parasite of stored grain beetles, especially *Tribolium castaneum* and *T. confusum*, isolated by Weiser (1953) [71], but has also been found in another Tenebrionidae of economic interest, *Alphitobius diaperinus* [72]. Like many protistan parasites, neogregarines appear to evade host recognition and do not induce melanisation or phagocytosis [12].

A recent study showed that coleopterans infected by an undescribed gregarine [73] and treated with sub-lethal insecticide levels had a lower survival relative to individuals just infected with gregarines or just exposed to insecticides [74]. This type of study demonstrates that additional stressors can act synergistically with parasites, resulting in a higher parasite burden and lower survival [74].

Within insect hosts, enteric eugregarines predominate. Moreover, they might be ubiquitous within insects, with each species perhaps having its own species of eugregarine parasite [12].

#### 2.2.3. Cryptosporidia

Cryptosporidia are known to cause cryptosporidiosis, an important waterborne human disease that manifests in dangerous diarrhoea. No treatment or effective therapy is currently available for this disease [49]. They are known to be epicellular parasites, originally found in the mouse gastric glands (*Cryptosporidium muris*) and intestine (*C. parvum*) [49].

Cryptosporidia are characterised by a specialised feeder organelle (located at the host–parasite interface and involved in the uptake of nutrients), which closely resembles the attachment site of some gregarines (mucron or epimerite) [49]. Like eugregarines, Cryptosporidia are considered as extracellular parasites. Despite these similarities, the traditional classification of Cryptosporidia within the coccidians has been rejected based on both ultrastructural and genomic differences. One of the latest phylogenomic analyses places the Cryptosporidia as a sister group to both the ‘core apicomplexan’ and gregarines [48].

##### Morphology, Host Range and Life Cycle

Cryptosporidia are monoxenous parasites and complete their development within a single host [49]. Forty species have been described, virtually all from vertebrates [75]. The infection starts when a host ingests an oocyst that releases sporozoites within the digestive tract. The sporozoites attach themselves to the host cell and are enveloped by the cell’s membrane, which creates an interface between the cell and the feeder organelle, allowing the parasite to steal nutrients from the cell. Cryptosporidian parasites then go through asexual and sexual reproduction, which both have the potential for autoinfection, leading to multiplication within the host and persistent infections. Heavily infected hosts release numerous oocysts in their faeces. The oocysts are fully infectious when excreted and are spread via horizontal transmission (host to host) and indirectly as waterborne or food-borne pathogens. The environmentally resistant ‘oocyst’ stage measures 4 to 8 µm in diameter and is characterised by a suture that was caused by the release of sporozoites.

##### Interactions with Hosts

Interestingly, although these parasites are known as pathogens of vertebrates, they are found on/in insect hosts, suggesting that these could serve as mechanical vectors. Some insects tend to have a phoretic association with *Cryptosporidium* parasites and can carry infective oocysts on their external surfaces and in their guts, even species that are supposed to be edible, such as *Tenebrio molitor*, revealing a potential human health problem in the consumption of insects [76,77,78,79].

### 2.3. Ciliates

The ciliates, like the Apicomplexa, are part of the Alveolata, which are unicellular organisms that live freely or as parasites and are characterised by the presence of cortical (outer) alveoli. Ciliates, as their name suggests, possess hair-like organelles called cilia and a complex oral apparatus, with a cytostome (specialised cells for phagocytosis), a cytopharynx and a digestive vacuole. They are common in freshwater and are characterised by a basal body composed of kinetosomes. The ciliates associated with insect hosts are typically commensals, and the few species that are entomopathogenic are able to penetrate the host cuticle and invade the tissues [80].

*Balantidium* spp. is a commensal ciliate that inhabits the digestive tracts of insects, especially cockroaches and termites. Other ciliates, such as *Rhabdostyla* spp., are commensal ectoparasites of several invertebrates, including insects [81].

#### Morphology, Host Range and Life Cycle

The two known entomopathogenic ciliate taxa are Hymenostomatida and Tetrahymenidae. Those ciliates are mostly known to cause infection and ciliatosis in dipteran hosts, such as mosquitoes and black flies, which can be infected by *Tetrahymena* spp. or *Lambornella* spp. [80]. Free-living stages are pyriform to ovoid and range from 40 to 80 µm in length. Recent molecular studies of ribosomal DNA showed the genus *Lambornella* to fall within the *Tetrahymena* genus [82,83]. Nevertheless, no formal revision of the genus has been conducted since.

### 2.4. Chlorophyta

The genus *Helicosporidium* sp. is the first and only known group of insect-pathogenic green algae [84]. These parasites are known to absorb nutrients from the insect host haemolymph [85]. Helicosporidia are characterised by their cyst stage that encloses three ovoid cells and a single elongate filamentous cell, protected by a pellicle [86]. They are closely related to the green algae *Prototheca*, which are the causative agent of protothecosis, a disease found in immunocompetent and immunosuppressed patients [87,88].

To date, a single species, *Helicosporidium parasiticum*, has been described by Keilin (1921) [89], and this may be responsible for all the insect infections induced by Chlorophyta as no morphological differences have been observed between isolates [90,91]. However, there is some genetic diversity between different *Helicosporidium* spp., with isolates from coleopterans more closely related to each other than to those extracted from dipterans [88]. *Helicosporidium* spp have been isolated from or transmitted to many invertebrates, including Coleoptera, Diptera and Lepidoptera [91,92,93,94], and metagenomic studies suggest that they are likely ubiquitous in lentic environments.

#### Morphology, Host Range and Life Cycle

**Figure 5:**
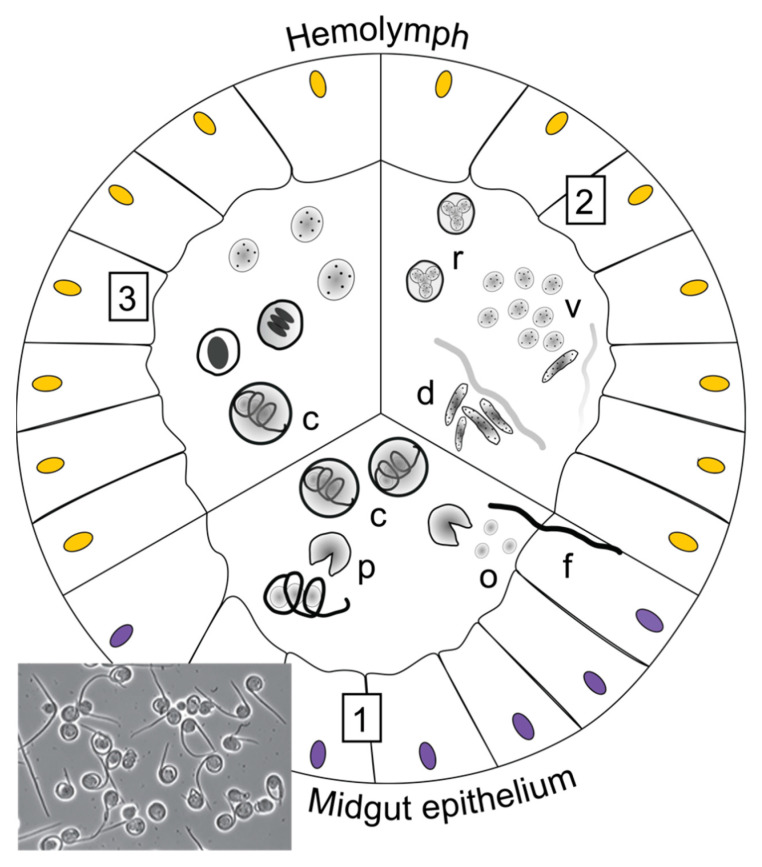
Generalised life cycle of *Helicosporidium parasiticum*. (**1**) Ingestion of cysts (c), which disrupt into the midgut, leaving an empty pellicle (p), and release filamentous cells (f) and ovoid cells (o) (three per cyst) that will be digested. The invasive filamentous cells penetrate the midgut epithelium and enter the haemocoel. (**2**) Within the haemolymph, filamentous cells undergo a transformation (swelling and reduction in size [86]) with a nuclear division. From here, new progeny cells (4 elongated rod-shaped daughter cells (d)) are formed and released from the filamentous cell pellicle. The elongated cells (d) then divide into 8 oval vegetative cells (v), which undergo further replication themselves (r). The replication of vegetative cells is also called autosporulation, where each cell can produce 2, 4 or 8 cells per mother cell [86]. Vegetative cells contain mitochondria, Golgi apparatus, a single nucleus, and dark granules in the cytoplasm [86]. (**3**) Eventually, vegetative cells differentiate into mature cysts (c). Phase contrast light micrograph shows disrupted cysts releasing filamentous and ovoid cells (reproduced with permission from Lange and Lord 2012 [12] Elsevier Science & Technology Journals).

Infection starts when the host ingests an infective cyst (Figure 5). The cyst is digested within the midgut and releases both ovoid cells (three) and a filamentous cell. The invasive filamentous cells penetrate the midgut epithelium and enter the haemocoel [86]. The filament cell possesses barbs at its surface, which might facilitate the passage through the epithelial layer to the haemolymph [85]. Within the haemolymph, Helicosporidia undergo multiple cycles of vegetative replications. Like the cyst stage, vegetative cells are non-motile and are enclosed within a pellicle that can contain two, four or eight daughter cells [85,95]. Eventually, the host haemocoel and haemolymph is colonised by massive numbers of vegetative cells, some of which differentiate into mature cysts [86]. It is still unclear if cyst morphogenesis is initiated intracellularly within haemocytes or extracellularly in the haemolymph, but it is hypothesised that cyst differentiation requires host-derived signals [86]. Newly formed cysts were observed after 5 to 6 days of infection within lepidopteran hosts, and measure around 5 µm [86].

*Helicosporidium* can be cultured in the lab in cells [85]. Its cysts can eventually be harvested, purified and can then be experimentally dehisced with larval digestive fluids from different lepidopteran species [85]. This could potentially represent a system for further investigation of the biology of these curious parasites.

#### Interactions with Hosts

Helicosporidia can have detrimental effects on hosts, such as reduced robustness and moderate mortality [12]. Bläske and Boucias (2004) treated early instar noctuids with *Helicosporidium* spp. And found that, while most pupated, only 20 to 30% of the pupated adults survived, and most of those had malformed wings and reduced longevity [96]. Additionally, infected *Galleria mellonella* (with the injection of 10^5^ cysts) all died at the larval pupal molt [96].

In experimentally infected *Spodoptera exigua* (Lepidoptera), *Helicosporidium* spp. Also induced a reduced fecundity and a transmission to progeny, with, however, no evidence of invasion of the reproductive tissue [96]. Infected *Helicoverpa zea* (Lepidoptera) showed up to 10^6^ cysts after 12 days of infection [86]. The infection could induce physiological changes, such as the degradation of the host’s cuticle, that would facilitate the release of cysts in the environment after the host death [86], but such effects have yet to be confirmed.

The massive multiplication of *Helicosporidium* vegetative cells in the host haemolymph indicates that those cells are able to avoid or suppress the cellular immune response of the host [86]. Indeed, infected *H. zea* did not show higher numbers of haemocytes compared to non-infected individuals; however, the underlying mechanisms are not yet known. In comparison, some fungi, such as *Beauveria* spp., can release proteases that reduce the haemocytes activity in *Galleria mellonella* [97].

Although *Helicosporidium* spp. have not been isolated from industrially reared insect species, their ubiquity in the environment and their ability to infect species of commercial interest under lab conditions, such as *Galleria mellonella* [96], makes them a potential threat to the insect rearing industry.

### 2.5. Euglenozoa

Euglenozoa are flagellated protists and part of the Discoba (Figure 1). Although typically free living, this group is home to some important parasite lineages within the Kinetoplastida, a group of organisms that are characterised by a kinetoplast (a large mass of mitochondrial DNA) and, specifically within the Trypanosomatida, a group of exclusively parasitic organisms [98]. The Trypanosomatids include serious human pathogens vectored by insects, particularly in the genera *Trypanosoma* and *Leishmania*. However, there is a large diversity of Trypanosomatids that are monoxenous and solely infect insects, and these have been relatively poorly studied [99,100]. Insect-infecting trypanosomatids have generally been considered benign; however, more recently, they have been implicated as pathogens that have negative impacts on pollinator health, particularly species within the genera *Crithidia* and *Lotmaria* [100,101]. It is unclear how these organisms cause pathogenic effects in their hosts, but one hypothesis is that they may carpet the hindgut, obstructing absorption or sequestering nutrients, resulting in a nutritional deficit for the host [102]. *Crithidia* species have not been identified as causing disease in commercially reared insects; however, they are increasingly reported to have low host specificity, not only infecting a broad range of insects but also multiple mammalian species [103].

### 2.6. Ichthyosporea

The Ichthyosporea, or Mesomycetzoea, is a clade of protists, closely related to animals within the Opisthokonta, which mostly parasitise aquatic animals and anurans [104]. There are at least 30 genera, and many species have a broad host spectrum. Ichthyosporea can persist in the environment thanks to a resilient spore stage. Because of their ability to persist in their environment, to have a broad host range and because of their virulence, ichthyosporeans are considered as emerging infectious pathogens [105].

The order Ichthyophonida (Cavalier-Smith 1998) was initially characterised by the presence of a motile amoeboid stage [106], and contains species isolated from insect hosts. Indeed, the Eccrinales family of the Ichthyophonida has been reported in association with insects that live in aquatic habitats [107]. Only one, undescribed, ichthyosporean, has been isolated from a terrestrial insect, *Tenebrio molitor* [108]. This isolate was found infecting the tissues beyond the gut lumen, including the fat body, testes and the ventral nerve system. The ichthyosporean symbiont found in *Tenebrio* did not show pathogenic effects. However, no bioassays were undertaken to determine if there is a difference between infected and uninfected colonies. The symbiont seems to persist through the generations even if the surfaces of the eggs are disinfected [108].

### 2.7. Microsporidia

The Microsporidia are obligate intracellular parasites related to fungi [21], which can infect a wide range of animal hosts from invertebrate to vertebrate species. Some species are also capable of infecting certain protist groups, such as ciliates and gregarines [109]. Microsporidia are characterised by a specific invasion apparatus (known as the polar tube), an extreme genome compaction, a lack of canonical mitochondria and ribosomes with rRNA genes of prokaryote-like size [21]. These parasites have been known to cause devastating microsporidiosis disease in mass-reared colonies of beneficial arthropods [11] and represent a serious potential threat for the insect rearing industry. One microsporidian species, *Nosema bombycis*, nearly destroyed the European silkworm industry during the 19th century [110]. To date, microsporidia are the most-studied protist-like insect parasites [111] and, in the last 150 years, around 1500 species in 200 genera have been described, with nearly half of them from insect hosts [112]. New microsporidia species are being discovered each year in different insect hosts, and more are being uncovered by molecular environmental sampling techniques [113,114,115,116,117,118,119,120]. Jaroslav Weiser, with his extensive experience researching microsporidia parasites, suggested that there might be a microsporidian in every living invertebrate species [121].

#### Morphology, Host Range and Life Cycle

Most microsporidia have a narrow host range and infect only one or several closely related host species. However, several microsporidia species show a broad host spectrum; those species could be defined as generalist parasites [21]. A specialist pathogen has a very fine host range (i.e., just one species or a few closely related species) and is highly adapted to its host, whereas a generalist pathogen has a broad host range and can infect hosts from different genera to families or orders. For example, species within the *Nosema*-*Vairimorpha* (Nosematidae) clade are known to infect a broad range of terrestrial insects, mostly Lepidoptera and Hymenoptera [122], with *Nosema bombycis* infecting more than 20 different Lepidopteran species in the environment surrounding silkworm rearing farms [123]. Similarly, the microsporidian *Paranosema locustae* can infect numerous different orthopteran host species [18].

**Figure 6:**
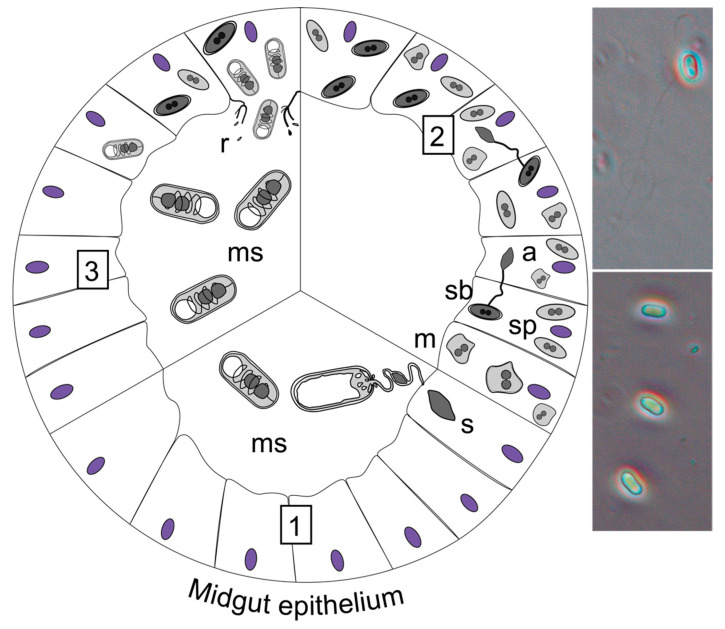
Life cycle of microsporidia based on *Vairimorpha apis* [112]. (**1**) A host ingests (binucleate) mature spores (ms) that germinate in the gut lumen and deposit the sporoplasm (s) directly into midgut epithelial cells. (**2**) The sporoplasm grows in size and matures into the first meront (m). Then, merogony occurs by binary fission and produces more meronts. At approximately 48 h post infection, some meront stages transform into sporonts (sp) that divide once by binary fission to form two sporoblasts (sb). This results in a primary binucleate spore (sb) characterised by a thin spore wall and short polar filament that germinates spontaneously within the cytoplasm of the epithelial cells. It is believed that this mechanism (autoinfection) serves to spread the parasite to adjacent epithelial cells (a). Other meronts within the cell continue to multiply and, after a number of divisions, enter into a second sporulation sequence. (**3**) Diplokaryotic sporonts (sp) divide once to produce two sporoblasts (sb), which, this time, mature into mature spores (ms). Mature spores have thick walls with long polar filaments. The infected epithelial cells become filled with spores and eventually rupture (r), releasing the spores into the gut lumen. These spores are expelled with the frass and contaminate the environment until ingested by a new host individual. Phase contrast light micrographs show microsporidian spores from *Gryllus bimaculatus* (top—germinated with polar tube showing, bottom—ungerminated. These spores measure ~ 6.6 × 3.3 µm).

Just like the other protists discussed above, microsporidia possess infectious spores that infect susceptible hosts (Figure 6). Infective spores have a spore wall and are generally very resilient to harsh environmental conditions. They can be ovoid, pyriform and rod-like, with typically a size between 1 and 12 µm and possess a unique filament that can be extruded when ingested by the host [124]. This ‘polar filament’ (or polar tube) is part of their unique infectious apparatus. When the spore is in the appropriate host and environment, the spore ‘germinates’, and, in some species, it is thought that this is induced by the host’s gut pH and digestive enzymes [125]. Thus, the polar tube is everted and pierces the host cell membrane (Figure 6). The sporoplasm (i.e., the spore content or “germ”) travels through this ‘injection tube’ and is inoculated into the host cell cytoplasm. At this moment, the parasitic and intracellular phase of the microsporidian life cycle begins.

Depending on the species, the life cycles are more or less complex [124]. Microsporidia parasites with simple life cycles undergo a single sporulation (production of new infective stages) within one host, whereas others undergo different sporulation sequences with intermediate stages and hosts [112]. Species from the Nosematidae family can have two sporulation sequences, a first one that produces ‘primary’ spores with thin spore walls and shorter polar tubes that spread the parasite to other host cells (autoinfection), followed by a second one that forms the infective and environmental stages with a thick spore wall [112,126].

The most common pathway of microsporidia transmission into a new host is through direct oral ingestion of infectious spores, which are found in food or liquids within the insect host’s immediate environment (e.g., soil, water, plant). Contamination of the environment with spores typically occurs when an infected host dies or when spores are released in faecal excrement or secretion. Horizontal transmission also occurs through cannibalistic feeding on moribund or dead infected individuals [112]. Vertical transmission, defined as the direct transfer of infection from parent to progeny, can also occur, with the transovarial (inside the egg cell) and/or transovum (on the surface of the egg) transmission as the main pathway [112].

Infections are typically systemic, but tissue tropism occurs in some species and, generally, the first developing stages are found in either the midgut’s lumen or epithelium [121].

#### Interactions with Hosts

**Figure 7:**
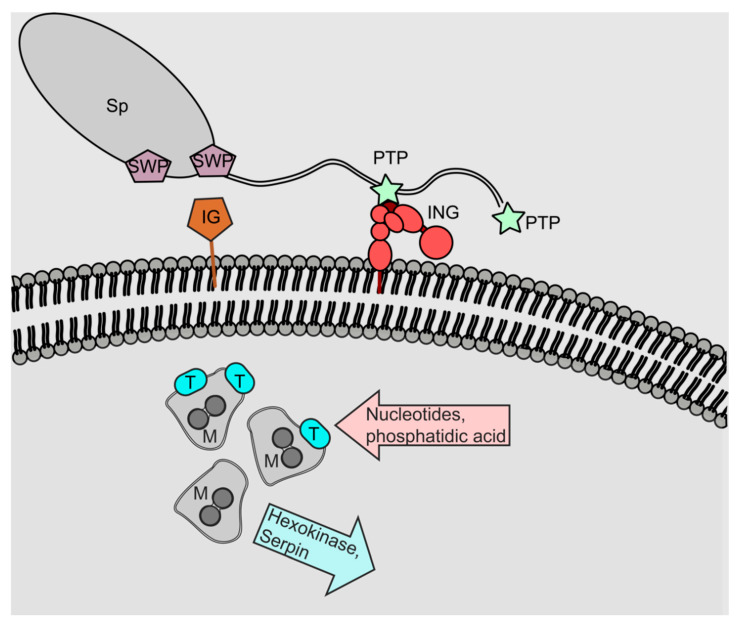
Simple schematic representation of insect host—microsporidia interactions during microsporidia infection. Spore wall proteins (SWP) on the spore (Sp) can bind to immunoglobulin (Ig) proteins present on the host cell membrane, such as the turtle-like protein found in *Bombyx mori* (BmTLP). Polar tube proteins (PTP) bind to other proteins, such as integrins (ING). Once the sporoplasm, now named meront (M), has entered the host cytoplasm, parasite proliferation begins. Microsporidia secrete hexokinases to influence host metabolism and use transporters (T) to uptake nutrients, including ATP and phosphatidic acid. The secretion of serpin proteins is hypothesised to inhibit the melanisation of proliferative stages.

As parasites of silkworms (*Bombyx mori*) and honeybees, the relationship between these insect hosts and their microsporidian parasites has been more intensively studied than other protist parasites, and research groups focusing on these particular hosts have started to unlock the interactions at the molecular level. Work in *Bombyx mori* has shown an interaction between a microsporidian spore wall protein (SWP26) and host cell membrane proteins, such as the immunoglobulin domain containing turtle-like protein. This interaction is hypothesised to facilitate host invasion [127] (Figure 7). The polar tube proteins also have a role in cell invasion, forming the main structure of the polar tube and attaching the host cell membrane via integrin proteins [128]. At least five polar tube proteins have been identified (PTP1–PTP5) [128,129].

Microsporidia generally have lost enzymes involved in energy and lipid metabolism and depend on hosts’ nutrients [130]. They have also lost their mitochondrial genomes and possess reduced mitochondria-related organelles known as mitosomes [131], which are unable to produce ATP. As a result of this reduction, microsporidia depend on the uptake of ATP and other substrates from their hosts [128]. Thus, meront stages possess transporters on their surface that obtain nutrients within the host cell, such as the nucleotide transporter family (NTT), that transport ATP and other nucleotides [132] (Figure 7). The transport of ATP can also be facilitated by the proximity of meront (proliferative) stages to the host cell’s mitochondria [133]. While the host is inaccessible, the environmental stages of microsporidia generate their own ATP through glycolysis [134]. In silkworms and honeybees, it has been shown that microsporidia impact and regulate the host’s metabolism, for example, resulting in higher food consumption in bees, or lower levels of ATP within silkworms [135,136]. Another key nutrient used by microsporidia in insect hosts seems to be phosphatidic acid [137]. In addition, proliferative stages can secrete enzymes such as hexokinase, which is thought to promote anabolic metabolism in the host [138]. Microsporidia can suppress one main insect host defence, the melanisation pathway, by inhibiting the enzyme Phenoloxidase (PO). It has been shown that *N. bombycis* secretes serpin proteins, which bind and impede the Prophenoloxidase Activating Proteinase and thus melanisation [139]. Moreover, it has been reported that microsporidia can prevent cell apoptosis via an unknown mechanism [140,141].

During infection, microsporidia can form xenomas, which result from specialised interactions between the parasite and the host cell [142]. Xenomas result in the fusion of infected cells (i.e., syncytial xenoma) or in the increased number of infected host cells (i.e., neoplastic xenoma) [143]. Xenomas provide benefits to both the parasite and the host, where they create a restricted niche with suitable conditions for the parasite to develop, protected from the host defence reactions. On the other hand, the host restricts the parasite to specific areas, preventing the spread to other more essential tissues [112].

The formation of xenomas by microsporidia could result from the manipulation of the host cell pathways to produce swollen cells [144]. It can be observed within silkworms infected by *Vairimorpha necatrix* but not by *N. bombycis* [145].

*N. bombycis* has been shown to cause a systemic and more virulent infection with transovarial transmission in silkworms and a LT50 ranging from 7 to 10 days [145]. It has been suggested that host specificity plays a role in the virulence of microsporidia, where species that have longer associations with specific hosts, such as *N. bombycis* in silkworms (*B. mori*), are better adapted to exploit their natural host than semi-permissive ones, such as *V. necatrix* [145].

Interestingly, the wax moth *Galleria mellonella* has been suggested as a resistant model against some microsporidia species. *Nosema—Vairimorpha* species are known to be generalist parasites of Lepidoptera, but *G. mellonella* was not susceptible or showed a low infection rate when experimentally infected with *V. ceranae* and *N. pyrausta* [146,147,148]. It is hypothesised that, because *G. mellonella* has a different diet compared to other phytophagous lepidopterans (composed mainly of wax and honey), it leads to a different, protective digestive system physiology [148].

The different host–parasite interactions and the effects of infection of the reviewed protists and microsporidia are summarised in Table 1.

## 3. Understanding the Diversity of Protistan Pathogens and Approaches to Detection

Whilst protists clearly have potential to have negative effects on hosts (summarised in Table 1), their diversity within farmed insects has not yet been fully explored. Identification of protist parasites has historically relied on microscopy and morphological identification in combination with knowledge of biological parameters, such as host specificity, tissues tropism, route of infection and the host spectrum [111,121]. Nowadays, molecular biology methods, such as polymerase chain reaction (PCR), barcoding and, more recently, high-throughput sequencing, are being widely applied for discovering novel protist lineage and to understand their contribution to microbiomes [7,9,153,154,155]. The 18S (small subunit) ribosomal RNA gene (18S) is the most extensively used genetic barcode for eukaryotic protist diversity studies [10,156,157,158,159]. The 18S genes encode the major RNA molecule of the small subunit of the ribosome and, as such, are conserved across all cellular life. In comparison to other gene markers that offer a finer resolution to study small eukaryote diversity, the 18S rRNA allows the exploration of eukaryotes as a whole and the design of ‘broadly targeted’ eukaryotic primers [9,10]. The 18S gene also contains nine relatively variable regions (named V regions) flanked by more conserved regions. Those variable and conserved regions, with different evolutionary rates, enable phylogenetic comparisons of distant and closely related taxa [160]. While numerous primers have been designed for 16S rDNA (the prokaryotic counterpart of the 18S) based on well-described V regions, few 18S rDNA primers are available to resolve eukaryotic diversity [161]. Analysis of the 18S rRNA gene has shown that the V4 and V5 regions are the most information-rich and suitable regions for microeukaryotic primer design and metabarcoding studies [10,161].

The application of metabarcoding to study host-associated microeukaryotes using the 18S barcode is challenging. Because, phylogenetically, animal hosts sit within protist diversity, primers that target all eukaryotes inevitably lead to co-amplification and read domination by host sequences [154,162]. To prevent this issue, ‘anti- metazoan’ primers have been designed to facilitate the amplification of parasitic protistan 18S templates from infected host tissues [163]. For example, a high-throughput metabarcoding sequencing method using these primers was carried out by del Campo et al. (2019) to study host-associated microeukaryotic diversity [162]. It consists of a two-step method where DNA extracts from the host are firstly amplified by 18S universal non-metazoan primers and secondly reamplified by broad primers that target the 18S V4 region to generate amplicons for sequencing [162].

A one-step approach is also possible with the use of fusion primers (i.e., primers with a degenerate section fused to a section of determined sequence—here, Illumina adaptors) coupled to Illumina sequencing, which is quicker and less prone to contamination; however, there is a greater risk of PCR inhibition [154]. Minardi et al. (2022) compared this one-step PCR, using the antimetazoan 574*F—UNonMet_ DB primers [7,161], to the above two-step PCR [154]. They found that the one-step anti-metazoan approach yielded proportionally more microeukaryotic amplicons than the two-step approach, depending on the sample nature and annealing temperature of the primers [154].

Although general eukaryotic primers and the general anti-metazoan primers mentioned above can capture much of the eukaryotic diversity, groups can be easily missed if they exhibit variation at the conserved primer site. One example of this is that the microsporidia are rarely picked up by general eukaryotic rDNA primers due to their accelerated rate of evolutionary change and unusual ribosome structure [21].

Parasites are subject to different selection pressures than free-living protistan relatives, which may lead them to have divergent genes sequences. This genetic divergence can mean that protist parasites are more likely to be missed by these broad primer approaches. It has been suggested that a large group-specific primer approach should be used to uncover diversity within specific clades [154]. However, to date, primer-specific primers only exist for certain protist groups [10].

Even if these ‘all diversity’ approaches may help to uncover the diversity of protists that may afflict commercially reared insect hosts, these do not help with the routine identification of protist pathogens within potentially infected colonies. In the future, there may be a need to accurately identify candidate etiological agents associated with new insect pathologies, some of which may be exacerbated by protist pathogens. In these situations, it will be crucial to have a set of PCR primers to assess the presence and prevalence of key protist pathogens. However, to date, some species of protist known to infect commercially reared insects still have no molecular data associated with them with which to generate diagnostic primer sets; for example, *Malamoeba locustae*, which infects reared orthopterans (Table 2). This highlights the need for molecular-based protist-focused microbiome surveys for these insects.

Currently, many high-throughput metabarcoding and sequencing methods used to study protists produce short reads with limited phylogenetic information, which complicates taxonomic identification [164]. Third generation sequencing, such as Nanopore sequencing, offers the possibility to sequence longer fragments with other advantages (e.g., portability and real-time sequencing). Most recently, Jamy et al. (2020) analysed soil protists by producing long sequence reads (~4,500 bp) while using broad eukaryotic primers that target a region covering the 18S and 28S (large subunit) rRNA genes [165]. The development of long-read metabarcoding comes with potential biases, with, for example, length biases occurring during the PCR as well as higher risks of chimera formation [153,166]. Nevertheless, long-read metabarcoding allows the sequencing of longer target regions, and linked information of the rRNA operons, 18S, ITS and 28S regions, can potentially be generated at the same time with the optimisation of long-range PCRs, which, eventually, may lead to the construction of more dense and robust reference phylogenies [153].

**Table 2:** Example of typical available molecular data for protists known to infect reared insects.

Parasite Group	Targeted Parasite	Accession	Host	Sequence Publication	Sample Tissue	Gene	Primers Used	Primer Specificity
Amoebozoa	*Entamoeba* sp.	LC259314	*Blaptica dubia*	[167]	Intestines	18S	01F/01R [167]	*Entamoeba* sp.
Ciliates	*Tetrahymena empidokyrea*	U36222	*Aedes* sp.	[168]	NI	18S	EukA/ EukB [169]	Eukaryotes
Coccidia	No coccidian isolates from insect hosts are available							
Cryptosporidia	No cryptosporidian isolates from insect hosts/vectors are available							
Gregarines	*Gregarina blattarum*	FJ459741	*Blattella germanica*	[62]	NI	18S	Lssu5/Lssu6 [170]	Eukaryotes
	*Gregarina cuneata*	FJ459744	*Tenebrio molitor*			18S		
	*Gregarina cloptoni*	FJ459742	*Tribolium freemani*			18S		
	*Gregarina niphandrodes*	AF129882	*Tenebrio molitor*	[171]	Intestines	18S	EukA/ EukB [169]	Eukaryotes
	*Gregarina polymorpha*	FJ459748	*Tenebrio molitor*	[62]	NI	18S	Lssu5/Lssu6 [170]	Eukaryotes
	*Mattesia* sp.	AY334569	*Solenopsis invicta*	[172]	Whole ants	18S	p71/p80 [172]	*Mattesia* sp.
Helicosporidia	*Helicosporidium* sp.	JN869301	*Dendroctonus mican*	[88]	NI	18S	MGF/ MGR [88]	*Helicosporidium* spp.
Ichthyosporea	*Ichthyosporea* sp.	JN699061	*Tenebrio molitor*	[108]	Nerve chord, testes and fat bodies	28S	500F/900R [108]	*Ichthyosporea* sp.
		JN699060				18S		
Microsporidia	*Anncaliia algerae*	AF069063	*Anopheles stephensi*	[173]	NI	18S	530f/580r and V1/ NOSr [173]	Microsporidia
	*Paranosema grylli*	AY305325	*Gryllus bimaculatus*	[174]	Fat bodies	18S	V1f/530r [175]	Microsporidia
	*Paranosema locustae*	AY305324	*Gryllus bimaculatus*			18S		
	*Paranosema whitei*	AY305323	*Tribolium confusum*			18S		
	*Tubulinosema kingi*	DQ019419	*Drosophila* sp.	[176]	NI	18S	HA3Bf/ HG2r [177]	Microsporidia
	*Tubulinosema ratisbonensis*	AY695845	*Drosophila melanogaster*	[178]	NI	18S	V1, 530f, 580r [179] and NOSr	Microsporidia
	*Tubulinosema suzukii*	MN631017	*Drosophila suzukii*	[114]	NI	18S	530f, 580r and 18f [180], Tn37 f and Tn562 r [114]	Microsporidia
	*Vairimorpha apis*	U26534	*Apis mellifera*	[181]	Ventriculus	18S	MICRO-F/ MCRO-R [182]	Microsporidia
	*Vairimorpha ceranae*	U26533	*Apis cerana*			18S		
	*Vairimorpha heterosporum*	L28973	*Spodoptera frugiperda*	[183]	NI	28S	580R [173]	Microsporidia
	*Vairimorpha necatrix*	Y00266	NI	[175]	NI	18S	V1F/530R [175]	Microsporidia

NI: Not indicated.

## 4. Conclusions and Future Research Needs

It is clear that protists commonly infect or are associated with insects that are currently being reared for food and feed (in the scope of the European regulation). However, the full diversity, the host range and potential to cause disease in this community have not yet been fully explored. Moreover, with the lack of ecological and physiological host range data and gene sequences, many protists’ host specificities are uncertain. Indeed, there may be synonymies among the described species in spite of differences in host spectrum and phylogeny.

Much of the literature on the protist communities in these insects predates the genomic era and, therefore, provide data to support morphological identification but not molecular identification. This is important as few researchers have an expertise in morphological identification that spans the diversity of protistan pathogens, although many have the skills to implement molecular identification tools across diverse taxonomic groups.

Although many protistan parasites cause few effects on their hosts in the wild, mass rearing conditions have the potential to favour parasite burden and to generate conditions that may lead normally symbiotic or benign infections to cause serious disease. Insects such as silkworms and honeybees have a long history of intensive commercial rearing, and microsporidia were recognised to be detrimental to these organisms very early on. However, it is only recently that eukaryotic pathogens, such as *Lotmaria* and *Crithydia* (Euglenozoa), have been investigated in *Apis mellifera* in order to understand their role in causing disease. This highlights the need for a greater awareness of protistan pathogens as the insect rearing industry grows. This will involve an increased effort to develop the molecular surveillance tools to monitor their association with insect ill health and, eventually, the development of host–parasite model systems and genomic resources to better understand the molecular basis of their pathogenicity.

## Figures and Tables

**Table 1:** Summary of host–parasite interactions (where data are available).

Parasite Lineage		Host–Parasite Interactions/Effects	
	Physical	Physiological	Biochemical Signals
Microsporidia 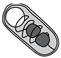	Attachment and piercing of the host cell by the polar tube [112] Dark melanic spots on the cuticle [112] Swollen abdomen [112] Reduction in host mobility [112]	Reduction in host fitness (higher mortality, reduced longevity, weight loss, lag in development) [112] Defacing of host fat bodies [112] Formation of hypertrophied tissues, ‘xenomas’, where spores proliferate (spatial sequestration) [142] Higher food consumption [135,136] Uptake of ATP through transporters [132] Inhibition of cell apoptosis [140,141]	Secretion of hexokinase [138] Secretion of serpin proteins (inhibition of melanisation) [139]
Gregarinida 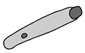	Anchoring and feeding structure (epimerite or mucron) [49] Swelling of the abdomen [149] Slower movements [149]	Reduction in host fitness (higher mortality, reduced longevity, weight loss, lag in development) [64,65]—can depend on host food intake [150] Increased susceptibility to other diseases or chemicals [74,149] Alteration in mating in relation to food intake [151] Reduction in fatty acid oxidation, with more lipids found around flight muscles, resulting in a more sedentary behaviour [152] Increase in trehalose (main blood sugar in insects) with a reduced response to insulin [152] Increase in fitness and longevity [66,67]	Insulin resistance potentially induced by excretory–secretory products from gregarines [152]
Amoebozoa 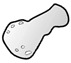	Enlargement of the Malpighian tubules [43] Disruption of tubules apical brush [36,41,42] Dark melanic spots within Malpighian tubule [12]	Increase in fluid secretion caused by tubule enlargement [43] Decrease in P-glycoprotein dependent detoxification caused by brush disruption [43] Change in unsaturated fatty acids levels of eggs [44] Reduction in feeding [31] Premature death and reduction in host fitness [31]	No data
Coccidia 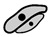	No data	Parasitophorous vacuole [12] Lag in host development [57]	No data
Chlorophyta 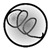	No data	Reduced robustness and higher mortality [96] Reduced fecundity [96]	Hypothesised immune system inhibition [86]
Euglenozoa 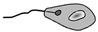	No data	Intestinal obstruction with nutritional deficit [102]	No data

## Supplementary Materials

The following supporting information can be downloaded at: https://www.mdpi.com/article/10.3390/insects13050482/s1, Supplementary Table S1. List of protist and microsporidia species naturally infecting reared insect hosts (from literature). Supplementary Table S2. Protist and microsporidia parasites used in successful experimental infection with reared insect species (from literature). References [184,185,186,187,188,189,190,191,192,193,194,195,196,197,198,199,200,201,202,203,204,205] are cited in the Supplementary Materials.
