# Supplementary material for: Protists in the Insect Rearing Industry: Benign Passengers or Potential Risk?
Source: Insects. 2022 May 21;13(5):482. doi: 10.3390/insects13050482 (PMC9144225; doi:10.3390/insects13050482)
Supplement: Supplementary file 1 [file insects-13-00482-s001.zip › insects-1686009-supplementary.pdf]

Supplementary Table S1. List of protist and microsporidia species naturally infecting reared insect hosts (from literature).

	Acheta domesticus	*Alphitobius diaperinus*	*Blaptica dubia*	*D. melanogaster*	*D. suzukii*	*Galleria mellonella*	*Gryllus bimaculatus*	*Locusta migratoria*	*M. domestica*	*Schistocerca gregaria*	*Tenebrio molitor*	*Zophobas morio*
**Microsporidia**												
**Nosematidae**												
Paranosema grylli							x [16]					
Paranosema locustae								x [18,46]				
Nosema sp.	x [78]							x [78]				
Vairimorpha apis						x [115]						
Vairimorpha ceranae						x [115]						
**Tubulinosematidae**												
Tubulinosema kingi				x [184]								

Tubulinosema ratisbonensis				x [178,185]								
Tubulinosema suzukii					x [114]							
**Incertae Sedis**												
Nosema galleriae						x [186]						
Microsporidia, indet.						x [46]					x [46]	
Microsporidium grylli							x [17]					
**Gregarines**	x [78]							x [78]			x [78]	
**Eugregarinorida**												
Gregarina acridiorum								x [187]				
Gregarina polymorpha											x [62,188]	
Gregarina niphandrodes											x [62,188]	
Gregarina garnhami										x [187]		
Gregarina cuneata											x [62,188]	
Gregarina steini											x [188,189]	
Gregarina alphitobii		x [190]										
Leidyana bimaculata							x [191]					
Eugregarines, indet.							x [151]	x [46]		x [46]	x [46]	x [46]
**Neogregarinorida**												
Schizogregarinina, indet.											x [46]	x [46]
Mattesia spp.								x [46]			x [46]	
Mattesia alphitobii		x [190]										
**Coccidia**												
**Eucoccidiorida**												
Isospora spp.	x [78]							x [78]			x [78]	
Adelina grylli							x [58]					
***Cryptosporidium* spp.**	x [78]							x [78]	x [192]		x [78]	
**Amoebozoa**												

Amoeba, indet.											x [46]	
Entamoeba spp.			x [167]					x [78]			x [78]	
Malamoeba locustae								x [46,193,194]		x [46,194]		
**Ciliophora**												
Balantidium spp.								x [78]			x [78]	

Supplementary Table S2. Protist and microsporidia parasites used in successful experimental infection with reared insect species (from literature).

	Acheta domesticus	*Blaptica dubia*	*D. melanogaster*	*Galleria mellonella*	*Gryllus bimaculatus*	*Locusta migratoria*	*M. domestica*	*Shistocerca gregaria*	*Tenebrio molitor*	*Zophobas morio*
**Microsporidia**										
**Nosematidae**										
Paranosema whitei				x [195]					x [195]	
Paranosema locustae								x [196,197]		
Vairimorpha necatrix				x [198]						
Vairimorpha heterosporum				x [199]						
Vairimorpha apis				x [147]						
Vairimorpha ceranae				x [147]						
**Tubulinosematidae**										
Tubulinosema spp.				x [146]						
Anncaliia algerae				x [199]						
Tubulinosema kingi			x [176,200–202]							
Tubulinosema ratisbonensis			[137]							
**Chlorophyta**										
Helicosporidium spp.				x [96]			x [96]			
Helicosporidium parasiticum				x [85,93]			x [85]			
**Gregarines**										
**Eugregarinorida**										
Gregarina garnhami								x [203]		
Gregarina niphandrodes									x [68]	
Gregarina polymorpha									x [204]	
**Neogregarinorida**										

Mattesia spp.				x [205]						
**Amoebozoa**										
Malamoeba locustae								x [43]		
